# One Health Insights into *Enterococcus*: Antimicrobial Resistance and Virulence in Companion Animals and Their Tutors

**DOI:** 10.3390/ijms27020654

**Published:** 2026-01-08

**Authors:** Joana Monteiro Marques, Beatriz Pita, Daniel Pinto, Maria Teresa Barreto-Crespo, Rosario Mato, Teresa Semedo-Lemsaddek

**Affiliations:** 1 Centre for Interdisciplinary Research in Animal Health (CIISA), Faculty of Veterinary Medicine, University of Lisbon, 1300-477 Lisbon, Portugal; 2 Associate Laboratory for Animal and Veterinary Sciences (AL4AnimalS), 1300-477 Lisbon, Portugal; 3 National Institute of Agricultural and Veterinary Research, IP (INIAV), 2780-157 Oeiras, Portugal; 4 NOVA School of Science and Technology (NOVA FCT), NOVA University Lisbon, 2829-516 Caparica, Portugal; 5 iBET—Instituto de Biologia Experimental e Tecnológica, 2780-157 Oeiras, Portugal; tcrespo@ibet.pt; 6 Instituto de Tecnologia Química e Biológica António Xavier (ITQB), 2780-157 Oeiras, Portugal; 7 UCIBIO—Applied Molecular Biosciences Unit, Department of Chemistry/Department of Life Sciences, NOVA School of Science and Technology, Universidade NOVA de Lisboa, 2829-516 Caparica, Portugal; r.labajos@fct.unl.pt; 8 Associate Laboratory i4HB—Institute for Health and Bioeconomy, NOVA School of Science and Technology, NOVA University Lisbon, 2829-516 Caparica, Portugal; 9 Biosystems & Integrative Sciences Institute (BioISI), Faculty of Sciences, University of Lisbon, 1749-016 Lisbon, Portugal

**Keywords:** *Enterococcus*, companion animals, healthy humans, One Health, antimicrobial resistance, virulence

## Abstract

*Enterococcus* spp. are opportunistic pathogens and commensals in humans and animals and are widely used as indicators of bacterial exchange, providing insights into antimicrobial resistance (AMR) and virulence dissemination within the One Health continuum. *Enterococcus* from healthy companion animals and their tutors were characterized to compare AMR profiles and virulence traits between hosts and within households in Lisbon, Portugal. Fecal samples (*n* = 45) were collected from 17 animals and 11 tutors. Enterococci were recovered from selective media, subjected to random amplified polymorphic DNA PCR (RAPD-PCR) and species identification, tested for antimicrobial susceptibility, and screened for virulence traits. Among animal isolates, 61% were *Enterococcus faecalis*, 29% *E. faecium*, and 10% *E. hirae*, whereas human enterococci comprised 52% *E. faecalis*, 35% *E. faecium*, 8% *E. hirae*, and 4% other species. Erythromycin resistance was identical in both groups (29%; Chi-squared test, *p* = 0.99). Ampicillin resistance was detected in all animal samples but was absent in human samples, whereas tetracycline and rifampicin resistance showed moderate host-specific patterns. Hemolytic activity was detected in 16% of animal and 31% of human isolates, all *cylA*-positive. Significant associations were observed between host origin and resistance to ampicillin and rifampicin, and between species and resistance to erythromycin and tetracycline. These findings suggest that companion animals can harbor, and potentially disseminate, AMR and virulence traits, reinforcing the need for One Health surveillance.

## 1. Introduction

Antimicrobial resistance (AMR) is among the most urgent global public health and development threats, estimated to have been directly responsible for 1.27 million deaths worldwide in 2019 and to have contributed to 4.95 million deaths [1]. In this context, the One Health approach, which integrates human, animal, and environmental health, is essential to understand, prevent and control AMR transmission within and across settings.

*Enterococcus* spp. are Gram-positive, ubiquitous bacteria, commensals of the gastrointestinal tract in humans and animals, while also occurring in diverse niches such as soil, water, and food. Enterococci are facultative anaerobic, catalase-negative, non-spore-forming cocci. In recent decades, they have emerged as opportunistic pathogens and are a major cause of serious healthcare-associated infections in humans, including urinary tract infections (UTIs), bacteremia, and infective endocarditis. Within the genus, *E. faecalis* and *E. faecium* together account for 90–95% of infections, whereas other species, such as *E. hirae*, *E. gallinarum* or *E. casseliflavus* are less frequently associated with infections [2]. This emergence is partly due to the remarkable adaptability of *Enterococcus* to adverse conditions and a variety of hosts, and propensity to acquire virulence and antibiotic resistance genes (ARGs) through horizontal transfer from cohabiting bacteria, including determinants that confer resistance to last-resort antibiotics such as vancomycin and linezolid [3,4,5,6]. Particularly, the vancomycin-resistant enterococci (VRE) cause severe multidrug-resistant infections and persistent colonization, which has led the World Health Organization to classify vancomycin-resistant *E. faecium* as a high-priority pathogen [7,8,9]. Healthy VRE carriers are common in the community, and despite being relatively high in human infections prior to the avoparcin ban, the prevalence has been decreasing in recent decades [10]. Although it is known that healthy animals are asymptomatic carriers of enterococci, infections in animals are associated with UTIs, otitis externa, peritonitis, endocarditis, and mastitis [11]. The prevalence of VRE was estimated to be 14.6% in companion animals around the globe, being more prevalent among dogs [12]. However, several studies have shown that VRE are rarely detected in healthy dogs [11,13,14], whereas resistant and multidrug-resistant enterococci are frequently observed [11,15,16]. The social role of companion animals is evolving, as they are increasingly considered integral members of households, and this close contact may facilitate the exchange of antimicrobial resistance genes between animals and humans [12]. Dogs and cats, particularly, have been reported as reservoirs of resistant bacteria in the gastrointestinal tract, posing a risk of transmission to their tutors [17]. This concern is amplified by the overlap in antibiotics commonly used in both human and veterinary medicine [12,17,18]. Previous One Health studies investigating gastrointestinal enterococci in companion animals and humans have shown differences in antimicrobial resistance patterns between hosts, while another study identified shared clones with common pathogenicity-associated traits, indicating potential dissemination within households [19]. Moreover, virulence determinants such as *agg*, *cylA*, *esp* and *gelE* are commonly identified among healthy animals [20,21,22,23] and humans [24].

The household environment represents a direct interface between humans and companion animals, providing ideal conditions for the circulation and potential exchange of microorganisms, including antimicrobial-resistant bacteria. Within this context, *Enterococcus* spp. are particularly relevant due to their occurrence, persistence, and prevalence across interconnected human, animal, and environmental settings, making them an ideal candidate for studying antimicrobial resistance from a One Health perspective. A One Health approach considers the interconnectedness of human, animal, and environmental health, aiming to understand how pathogens and resistance determinants can spread across these compartments. Despite the recognized role of *Enterococcus* spp. as sentinel organisms for antimicrobial resistance [2], the extent to which household sharing influences resistance patterns between companion animals and their human tutors remains poorly elucidated.

By collecting samples simultaneously from companion animals and their human tutors, this exploratory study applies a practical One Health design that captures interactions occurring at the human–animal interface in domestic settings. This paired sampling enables parallel characterization of *Enterococcus* strains circulating in both hosts and allows the identification of shared or host-specific traits. In doing so, the study explores how companion animal–human interactions may shape antibiotic-resistant and virulent *Enterococcus* populations within household settings in Portugal, emphasizing the importance of integrated surveillance and control strategies under the One Health framework.

## 2. Results and Discussion

*Enterococcus* isolates from paired household samples of companion animals and their tutors were analyzed to evaluate the occurrence and potential sharing of antimicrobial resistance and virulence traits within the framework of One Health surveillance. This exploratory study followed a cross-sectional household surveillance and was not designed as a longitudinal time-series study; therefore, no temporal dynamics analyses were performed.

### 2.1. Sampling and Microbial Isolation

Over five months, 45 fecal samples from healthy companion animals (*n* = 29) and their human tutors (*n* = 16) across five Lisbon households were processed for enterococcal isolation using Slanetz and Bartley agar (SBA) and vancomycin-supplemented SBA (SBAvan), followed by confirmation on Bile Esculin Azide agar. Vancomycin supplementation was applied to select potential vancomycin-resistant enterococci (VRE), given the clinical relevance of this resistance. However, even at a low concentration (6 µg/mL), vancomycin markedly reduced growth by imposing additional stress, which resulted in fewer samples with enterococcal colonies and therefore a lower number of isolates being recovered from supplemented medium.

Overall, a total of 166 enterococci were obtained, of which 78 originated from companion animals (66 from SBA, 12 from SBAvan), and 88 from humans (68 from SBA, 20 from SBAvan). The prevalence of enterococci in cat feces was markedly lower (65.2%) compared to dogs, where all samples were positive (100%). Other authors also reported this lower prevalence associated with enterococci from cat feces compared with dogs. Jackson et al. (2009) reported comparable findings, with enterococci being isolated from 60% of cats and 80% of dogs, although sampling occurred across different body sites [25]. Wada et al. (2021) focused on the prevalence of VRE in companion animals and found a prevalence of 12.3% in cats compared to 18.2% in dogs, also comparable to the enterococci obtained from vancomycin-supplemented media in cats and dogs in our study [12]. Regarding the human samples analyzed on the present research, all were positive for enterococcal growth.

### 2.2. Molecular Characterization

**Figure 1:**
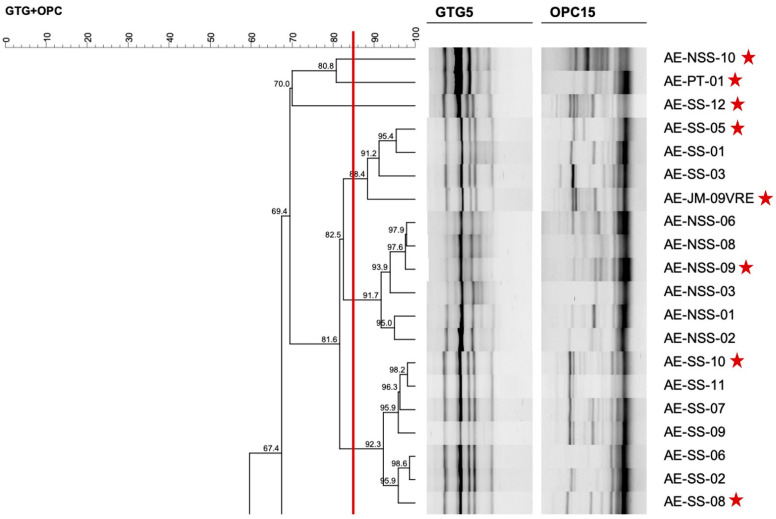
A section of the dendrogram created with RAPD-profiles of *Enterococcus* from humans, used to select genomically distinct isolates for further characterization. The red line corresponds to the reproducibility level based on replicate analysis, used as similarity cut-off value. Red stars indicate the enterococci chosen as representative of the microbial diversity.

After purification of single colonies, RAPD-PCR was performed using primers (GTG)_5_ and OPC15 in separate reactions. The resulting amplification profiles were used to assess microbial diversity and select genomically distinct enterococcal isolates for further characterization. In brief, the genetic diversity analysis by RAPD-PCR for companion animals and humans’ enterococci revealed similar or highly similar profiles among bacterial cultures selected from the same isolation plate, and in some cases between isolates from humans and animals within households, suggesting possible shared colonization. However, to ensure that subsequent analysis was based on overall diversity and not clonality, only genetically distinct isolates were selected from RAPD clusters, considering the reproducibility threshold of >85%, enterococcal source of origin (companion animals or humans) and household representativity. Figure 1 shows an example of a section of the dendrogram used for the selection of human-derived (tutors) enterococci.

Dendrogram analysis allowed the selection of a total of 79 enterococci, 31 from animals (17 from cats and 14 from dogs) and 48 from their human tutors. From the 31 isolates from animals, 24 were initially recovered from selective media and 7 from selective media with vancomycin supplementation. As for the 48 isolates from humans, 37 originated from selective media and 11 from vancomycin supplemented media. Selected enterococci were subsequently submitted to PCR for identification at genus and species levels; results obtained are presented in Table 1. Details on the representative isolates, their host distribution, and corresponding species are presented in Supplementary Table S1.

Overall, *E. faecalis* and *E. faecium* accounted for the vast majority of isolates in both groups. Among animal isolates, 61.2% (19/31) were identified as *E. faecalis*, 29% (9/31) as *E. faecium*, and 9.7% (3/31) as *E. hirae*. Although the higher prevalence of *E. faecalis* isolated from healthy companion animals is consistent with previous reports [25,26,27,28], other studies have found *E. faecium* equally prevalent, or even more common than *E. faecalis* in fecal samples in cats and dogs [11,21,23,29,30]. Regarding human isolates, 52.1% (25/48) were identified as *E. faecalis*, 35.4% (17/48) as *E. faecium*, 8.3% (4/48) as *E. hirae*, and 4.2% (2/48) as *Enterococcus* sp. (identified only at the genus level). The distribution of *Enterococcus* species identified in healthy human’ samples is also consistent with previous reports [31].

Although *E. faecalis* was the predominant species in both hosts, it was more frequent among companion animals, whereas *E. faecium* was comparatively more common in tutors. Nevertheless, the distribution of *Enterococcus* species did not differ significantly between isolates from companion animals and their human tutors (Fisher’s exact test, *p* = 0.712), indicating that species composition was generally similar across host origins.

### 2.3. Antimicrobial Resistance

#### 2.3.1. Antimicrobial Susceptibility Testing

Antimicrobial susceptibility testing was performed on 79 enterococcal isolates from animals and humans through the disk diffusion method, using 16 antimicrobials clinically relevant in veterinary and human medicine.

**Figure 2:**
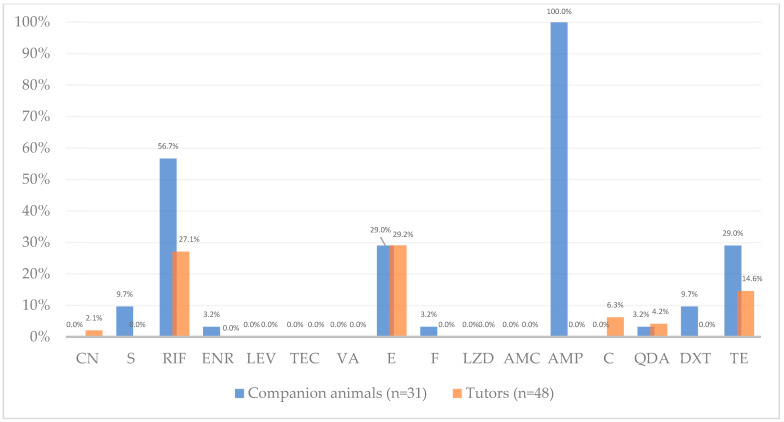
Antimicrobial resistance frequency (%) among *Enterococcus* from companion animals (blue, n = 31 isolates) and their human tutors (orange, n = 48 isolates). Percentages refer to proportions of isolates resistant to each antibiotic; exact counts are reported in Table 2. Antibiotics tested, by alphabetic order of classes: CN—gentamicin, S—streptomycin, RIF—rifampicin, ENR—enrofloxacin, LEV—levofloxacin, TEC—teicoplanin, VA—vancomycin, E—erythromycin, F—nitrofurantoin, LZD—linezolid, AMC—amoxicillin-clavulanic acid, AMP—ampicillin, C—chloramphenicol, QDA—quinupristin-dalfopristin, DXT—doxycycline, TE—tetracycline. Note: QDA resistance results exclude *E. faecalis* as this species is intrinsically resistant to this antibiotic.

Enterococci were classified as resistant, intermediate and susceptible, according to the breakpoints defined by CLSI [32]. The distribution (frequency and percentage) of resistant, intermediate, and susceptible *Enterococcus* isolates from companion animals and their human tutors is shown in Table 2, while Supplementary Table S1 provides a detailed summary of the antibiotic resistance results and phenotype profiles for each isolate. In addition, Figure 2 provides a visual comparison of *Enterococcus* resistance percentages between host groups.

Antimicrobial susceptibility testing revealed distinct resistance profiles between companion animals and human enterococci, except for erythromycin. Erythromycin (macrolide) resistance was moderate and identical in both groups (~29%; Chi squared test, *p* = 0.99), occurring in isolates from both companion animals and humans in all households (Table S1 in Supplementary Materials). This equal resistance rate in both groups is likely associated with the overlapping use of macrolides in both human and veterinary medicine, together with the ability of mobile genetic elements to mediate resistance transfer between hosts [19]. This resistance frequency is consistent with other studies supporting that macrolide resistance, and resistance genes, particularly *erm*(*B*), are widely distributed across human and animal enterococci [19,21,23]. Previous studies conducted in companion animals under veterinary care in Portugal and Brazil reported erythromycin resistance rates of 53% and 68%, respectively [33,34].

On the other hand, a striking divergence in ampicillin (penicillin) resistance was observed between groups, to which all animal isolates were resistant (100%, 31/31), in contrast with total susceptibility in human enterococci. Accordingly, statistical analysis revealed a significant association between host origin (companion animals vs. tutors) and resistance to ampicillin (Fisher’s exact test, *p* < 0.001, adjusted *p* < 0.001). This prevalence was higher than previously reported in animals, but lower than previously observed in humans [11,19,31]. In a similar study, Iseppi et al. (2020) reported higher ampicillin resistance in cats compared to humans, while dogs showed resistance levels similar to those observed in human isolates [35]. This difference could be influenced by distinct selective pressures between veterinary and human medicine. In companion animals, the empirical use of ampicillin in veterinary hospital or clinical settings for common infections may increase exposure of commensal *Enterococcus* populations. In contrast, in humans, ampicillin is less frequently prescribed and is reserved for enterococcal infections exhibiting high-level resistance to other antimicrobials [36]. Notably, resistance to amoxicillin-clavulanic acid, a β-lactam often prescribed to outpatients as an alternative to ampicillin in both veterinary and human medicine, was absent in both host groups. This susceptibility detected by disk diffusion should be interpreted cautiously, as aminopenicillin susceptibility in enterococci is usually inferred from ampicillin results. As antimicrobial therapy within six months prior to sampling was excluded through informed consent verification, and no further data on antibiotic use were available, this interpretation should be considered hypothetical and warrants further investigation. Ampicillin resistance in *Enterococcus*, particularly *E. faecium*, is associated with increased production of penicillin-binding protein 5 (PBP5) or alterations in the C-terminal region of *pbp5* gene [37]. However, we did not perform *pbp5* sequencing nor ampicillin MIC testing in this study. We therefore present these results as phenotypic observations and recommend follow-up MIC determination and *pbp5* sequence analysis to determine the molecular basis of the observed resistance. Nevertheless, considering our results, it is recommended to reduce the empirical use of ampicillin in veterinary clinical practice.

Resistance to rifampicin (ansamycin) was detected in enterococci from both companion animals and their tutors in four of the five (80%) households in study (Table S1 in Supplementary Materials). Resistance was twice as high in companion animal isolates (56.7%, 17/30) compared with humans (27.1%, 13/48). Supporting this observation, statistical analysis revealed significant association between host origin (companion animals vs. tutors) and resistance to rifampicin (Chi-squared test, *p* = 0.001; adjusted *p* = 0.05), with animal enterococci showing higher resistance than tutors. The difference is likely related to historical differences in usage practices in veterinary and human medicine. Although rifampicin use in animals is nowadays limited in Portugal and across the EU [38,39], the comparatively higher resistance rates observed in companion animal isolates may reflect historically less regulated antimicrobial use and insufficient surveillance [40], which has likely contributed to the selection and persistence of resistance in animal reservoirs. A study conducted in Portugal found high rifampicin resistance among dogs (47.9%) and moderate levels among cats (12.4%). The authors reported that resistance was approximately three times higher in dogs than in cats and occurred more frequently in animals with mixed habitat types (indoor/outdoor) [33]. Similar patterns were observed in the present study, with rifampicin resistance detected in 78.6% of dogs (with outdoor access) and 31.2% of cats (indoor access only). Other studies in Brazil reported high rifampicin resistance in enterococci from hospitalized companion animals, with frequencies of 80.7% [34] and 67.8% [18]. From an environmental perspective, several studies have observed high rifampicin resistance among enterococci isolated from vegetables, water, soil, sewage and clinical infections, with prevalence ranging from 50% to over 80% across the US, Spain, and Portugal [41,42,43]. Such widespread environmental resistance distribution may contribute to the elevated resistance observed in companion animals, particularly those with outdoor exposure. Given the high prevalence of rifampicin resistance detected in companion animals, rifampicin use in veterinary clinical practice should be further restricted and carefully monitored to limit additional selective pressure in veterinary settings and across One Health interfaces.

In humans, rifampicin usage is also restricted to specific indications and only administered in synergy with other antimicrobials, such as daptomycin [44], which may comparatively reduce selective pressure for resistance development. Notwithstanding possible usage differences, acquired resistance to rifampicin in enterococci remains common, with 79% of resistance in 71 clinical isolates reported by a prior study, and it is often associated with chromosomal mutations in the *rpoB* gene (reviewed by [36]). Acquired resistance in both animals and humans may result from commensal enterococci being exposed to rifampicin during the treatment of non-enterococcal infections [36]. However, a meta-analysis reported that although generally high among clinical enterococci, rifampicin resistance was lowest in Europe (38.9%) [45].

Similarly, tetracycline resistance was relatively high among companion animal enterococci (29%, 9/31) but lower in those from humans (14.6%, 7/48). No significant associations were found between host origin and resistance to tetracycline (Chi-squared test, *p* = 0.11, *p* adjusted = 0.21). Resistance was detected in both hosts in three of the five households (60%) and exclusively in animals in the remaining two (Table S1 in Supplementary Materials). In contrast, doxycycline resistance was observed only in companion animal isolates, with a low frequency (9.7%, 3/31). Only 50% of the tetracycline resistant enterococcal isolates exhibited intermediate or resistant phenotypes to doxycycline. Differential susceptibility between these two antibiotics has been previously reported and can be explained by the distinct mechanisms underlying tetracycline resistance. In *Enterococcus*, this resistance is mainly mediated by ribosomal protection proteins such as *tet*(*M*), which act with variable effectiveness across tetracycline derivatives [13,46,47]. The higher binding affinity, lipophilicity and intracellular penetration of doxycycline may contribute to its sustained efficacy [48]. Consistent with our observations, a meta-analysis indicates that clinical enterococci generally exhibit lower resistance to doxycycline than to tetracycline, with pooled resistance estimates of 31% (95% CI: 12.6–59.3%) and 67% (95% CI: 56.8–75.1%), respectively [45]. Similar patterns have been observed in other species, where doxycycline susceptibility was frequent despite tetracycline resistance [49]. Beyond these antibiotic-specific differences, previous studies have also reported higher tetracycline resistance among enterococcal isolates from animal compared to those from human origin [19,31].

Resistance to high-level gentamicin and streptomycin (aminoglycosides) was low in both groups, with gentamicin resistance only detected in human isolates with low percentage (2.1%, 1/48), and slightly higher streptomycin resistance observed only among human tutors (9.7%, 3/31). No significant associations were found between host groups and gentamicin (Fisher test, *p* = 1, *p* adjusted = 1) and streptomycin (Fisher test, *p* = 0.06, *p* adjusted = 0,13). High-level aminoglycoside resistance is generally uncommon in dogs [11], while in humans it varies according to geographic location and the specific setting [24]. In Portugal, a One Health related study also reported 2% gentamicin resistance in enterococci from stool samples of healthy humans [19].

Enrofloxacin (fluoroquinolone), nitrofurantoin (nitrofurans), chloramphenicol (phenicol) and quinupristin-dalfopristin (streptogramins) resistance were generally low in both groups, with slight differences between animals and human enterococci. No resistance was detected to levofloxacin (fluoroquinolone), teicoplanin (glycopeptide), linezolid (oxazolidinone), or amoxicillin–clavulanic acid (penicillin) in either group.

Although enrofloxacin is not routinely used against *Enterococcus* spp. in veterinary or human medicine and no CLSI or EUCAST breakpoints are available, it was included in our susceptibility panel for epidemiological purposes. Enrofloxacin is a fluoroquinolone widely used for Gram-negative infections in animals, and its main active metabolite, ciprofloxacin, is a critical antimicrobial in human medicine. However, enrofloxacin is not approved for use in human medicine, and the absence of resistance reported in this study is consistent with the lack of selective pressure within commensal reservoirs within this population. No levofloxacin resistance was detected in either animals or humans, indicating a low level of fluoroquinolone resistance, consistent with some previous studies [19,23], but not in line with others [21,22].

Quinupristin-dalfopristin resistance was not considered for isolates identified as *E. faecalis*, which are known to be intrinsically resistant to this antibiotic [19]. The absent resistance observed among human *E. faecium* isolates is consistent with the low quinupristin-dalfopristin resistance prevalence among humans (2%) found by Poeta et al. (2006), although the authors reported higher resistance in companion animals (15%) compared to the present study [19]. Conversely, Almeida-Santos et al. (2025) observed high resistance to quinupristin-dalfopristin in *E. faecium* and *E. lactis* from the healthy human gut in 2001 (64%) and 2022 (36%) [31].

Resistance to linezolid, also a last-resort treatment for severe infections in both humans and animals, was absent in all enterococci under study. This result is consistent with its limited use in both groups and might also reflect the small sample size.

Glycopeptide resistance was overall not detected. A high percentage of vancomycin resistance (51.6%, 16/31) was initially reported by the disk diffusion method in animal isolates (Table 2). However, as indicated by CLSI guidelines [32], this resistance was subsequently confirmed by dilution methods such as agar dilution and determination of Minimal Inhibitory Concentration (MIC). In these assays, no resistance was detected, with MIC values below 4 µg/mL and one intermediate result (MIC = 8 µg/mL); hence, all enterococci previously reported as resistant were reclassified accordingly (as presented in Figure 2). A recent study reported comparable findings in enterococci isolated from feces of healthy dogs and urine of dogs with urinary tract infections, in which 13.7% were initially identified as vancomycin-resistant, but none were confirmed by MIC testing [11]. A few biological reasons and methodological limitations can be behind this discrepancy between methods. First, the technical limitations of disk diffusion for vancomycin in enterococci has been highlighted by CLSI and other commissions, due to glycopeptide resistance mechanisms which may lead to low-level resistance resulting in misclassification by disk diffusion. Some isolates can present zone inhibitions near the resistance threshold due to growth delays when exposed to vancomycin, or to resistant subpopulations (heteroresistance), which may result in inaccurate resistance classification [50]. Other reasons include methodology-associated variability (including media composition), which may be overcome by MIC determination. Moreover, high error rates associated with the disk diffusion method have been reported by other authors [51,52]. In addition, enterococci isolated from selective media supplemented with vancomycin in the present study (screening concentration) were generally not confirmed as resistant, and from those initially reported as resistant by disk diffusion, only two were obtained from SBAvan. As for human isolates, no vancomycin resistance was detected (Table 2, Figure 2). Thus, the absence of glycopeptide resistance observed in the present study suggests a low likelihood of vancomycin-resistant enterococci transmission between companion animals and tutors, but ongoing monitoring across One Health domains remains advisable due to vancomycin’s clinical importance. Other studies also reported absence or low vancomycin resistance in enterococci from animals and humans [13,19,21,23,24,27,28,30,53,54,55].

**Figure 3:**
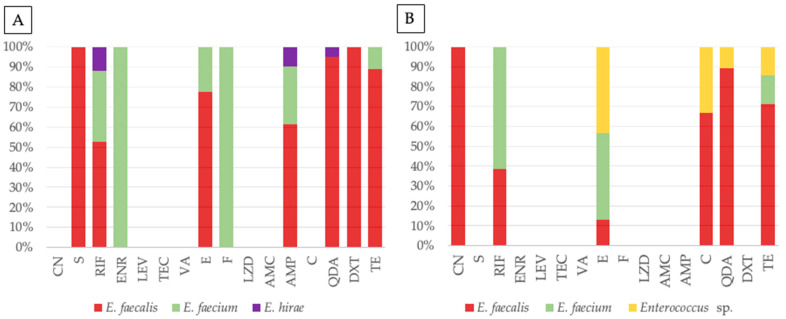
Antimicrobial resistance profiles of *Enterococcus* from companion animals (**A**) and tutors (**B**), with stacked bar plots representing the percentage distribution of resistant isolates for each antibiotic tested, grouped by species: *E. faecalis* (red), *E. faecium* (green), *E. hirae* (purple), and *Enterococcus* sp. (yellow). Antibiotics tested, by alphabetic order of classes: CN—gentamicin, S—streptomycin, RIF—rifampicin, ENR—enrofloxacin, LEV—levofloxacin, TEC—teicoplanin, VA—vancomycin, E—erythromycin, F—nitrofurantoin, LZD—linezolid, AMC—amoxicillin-clavulanic acid, AMP—ampicillin, C—chloramphenicol, QDA—quinupristin-dalfopristin, DXT—doxycycline, TE—tetracycline.

The frequency of antimicrobial resistance in *Enterococcus* isolates from companion animals and their human tutors, by species, is represented in Table 3. Resistance profiles were species-dependent, with *E. faecalis* and *E. faecium* acting as the primary reservoirs of antimicrobial resistance, and *E. faecium* exhibiting more targeted resistance traits in companion animals and humans. Figure 3 displays the antimicrobial resistance to each antibiotic distributed by *Enterococcus* species, in companion animals and human tutors. In animals (Figure 3A), resistance was widely distributed across *E. faecalis* and *E. faecium*, with some *E. hirae* being resistant to rifampicin, ampicillin and quinupristin-dalfopristin. Enrofloxacin and nitrofurantoin resistance was identified only in animal *E. faecium*. In contrast, human isolates were dominated by *E. faecalis* and *E. faecium*, with other enterococci (not identified at the species level) being more noticeably resistant to chloramphenicol, tetracycline, quinupristin-dalfopristin and erythromycin. Rifampicin and erythromycin resistance were mainly identified in human *E. faecium*. Considering all results, statistical analysis revealed a significant association between species allocation and resistance to erythromycin (Fisher’s exact test, *p* = 0.031) and tetracycline (Fisher’s exact test, *p* = 0.037).

To estimate the overall association between enterococci from companion animals and human tutors regarding antimicrobial resistance, odds ratio was calculated for each antibiotic, and a mixed-effects logistic regression was fitted. Odds ratio analysis suggested that tutors had higher odds of resistance than animals only for gentamicin (OR = 1.99, 95% CI: 0.07–50.4, *p* = 1) and chloramphenicol (OR = 4.85, 95% CI: 0.24–97.1, *p* = 0.275), as resistance was observed exclusively among tutor isolates for these antibiotics, but the effect was not significant in both cases, reflecting the small number of enterococci under study. Overall, both models showed that tutor isolates had significantly lower odds (~80%) of resistance compared with companion animals (OR = 0.20, 95% CI: 0.13–0.33, *p* < 0.001). This global model included host origin (companion animals vs. human tutors) as a fixed effect, antibiotics as covariates and isolate as a random effect.

When analyzing antimicrobial resistance by household origin, an association was observed for ampicillin (Fisher’s exact test, *p* = 0.035). However, this association was not considered significant after correction for multiple testing (adjusted *p* = 0.567). No other antibiotics showed significant associations with household origin, so overall there is no robust evidence of household-level clustering of antimicrobial resistance.

Multidrug resistance (MDR) is defined as the acquired non-susceptibility to at least 1 antimicrobial belonging to 3 or more antimicrobial classes [56]. As only acquired non-susceptibility is considered, quinupristin-dalfopristin intermediate and resistance results were not taken into account for *E. faecalis*, as this species is intrinsically resistant to this antibiotic [19]. Accordingly, multidrug resistance (MDR) was identified in 90.3% (28/31) of animal enterococcal isolates compared with 33.3% (16/48) of tutor enterococci. Among species, *E. faecium* had the higher percentage of MDR, with 57.7% (15/26), followed by *E. faecalis* with 54.5% (24/44). The higher levels of AMR and MDR among *E. faecium* have been previously reported in enterococci from companion animals [21,57]. These results are particularly concerning in a One Health context, as they highlight the potential role of companion animals as reservoirs and disseminators of multidrug-resistant strains, including *Enterococcus*, within household environments. Shared environments, including households, veterinary clinics and public spaces represent critical interfaces linking human, animal and environmental health, facilitating bacterial and antimicrobial resistance exchange. Thus, it is urgent to strengthen integrated surveillance across veterinary and human sectors and implement targeted intervention strategies to mitigate antimicrobial resistance dissemination throughout the One Health continuum.

#### 2.3.2. Screening for Resistance Genetic Determinants

The resistance phenotypes observed for erythromycin, high-level gentamicin, tetracycline/doxycycline, and vancomycin were confirmed by multiplex PCR, by the presence of the genes *erm*(*B*), *aacA-aphD*, *tet*(*M*), and *vanA*/*vanB*/*vanC*, respectively. The aim was to provide a targeted phenotypic–genotypic overview of the most epidemiologically relevant determinants within the framework of household-level One Health surveillance, rather than an exhaustive genomic screening of all possible resistance genes. Table 4 presents the phenotype–genotype comparison of antimicrobial resistance in *Enterococcus* isolates from companion animals and their tutors, and Supplementary Table S1 lists the specific resistance genes identified for each isolate.

Resistance gene screening revealed the presence of *aacA-aphD* in the single gentamicin-resistant tutor enterococci, supporting a total correlation between the phenotype and genotype. In case of *ermB*, it was present in 30.4% (7/23) of erythromycin-resistant isolates, and *tetM* in 31.2% (5/16) of tetracycline-resistant enterococci (mainly from companion animals). A study investigating the prevalence of the resistance genes *aac*(*6′*)*-aph*(*2″*) (gentamicin), *erm*(*B*), and *tet*(*M*) found that, for the first two genes, all human and animal *E. faecalis* isolates carried the gene, with complete concordance between phenotype and genotype, whereas in *E. faecium*, the prevalence of the corresponding resistance gene among resistant isolates was only 40% in humans [19]. The prevalence of *tet*(*M*) found in this investigation is consistent with the results reported in that same study [19]. An 85% prevalence of the *erm*(*B*) gene among erythromycin-resistant enterococci has been reported by other authors, whereas our results show a comparatively lower prevalence [24]. However, the absence of the gene in phenotypically resistant isolates suggests that resistance may be mediated by other determinants, not included in the screening panel (e.g., *tet*(*L*), *tet*(*K*), *erm*(*A*), *erm*(*C*), *aph*(*3′*)*-IIIa*). No vancomycin resistance genes (*vanA*, *vanB*, *vanC*) were detected in this study, which is consistent with the absence of resistance reported by broth microdilution methods.

### 2.4. Virulence Traits

#### 2.4.1. Hemolytic Activity

Hemolytic activity was assessed on blood agar plates incubated under anaerobic conditions. These conditions were chosen to avoid oxidation and erythrocyte lysis caused by factors other than enterococcal hemolysin production, and because they provide a more reliable assessment of virulence relevant to human health [58]. Hemolytic activity (β-hemolysis) was observed in 20 of the 79 isolates (25.3%), identified by the presence of a clear halo, and the corresponding results for each isolate are detailed in Supplementary Table S1. Most hemolytic enterococci were recovered from humans (15/48; 31%), whereas companion animals accounted for 5/31 (16.1%). The majority of the hemolytic enterococci from humans were *E. faecalis* (80%, 12/15), with a small percentage of *E. faecium* (13.3%, 2/15), while all β-hemolytic isolates from companion animals were *E. faecalis* (100%). These findings are consistent with those of Kubašová et al. (2017), who have also reported all β-hemolytic as *E. faecalis*, although the prevalence of hemolysis in their study (8.2%) was notably lower [22].

#### 2.4.2. Screening for Virulence Genetic Determinants

Genetic virulence determinants were screened by multiplex PCR for *agg* (aggregation substance), *cylA* (cytolysin activator), *esp* (enterococcal surface protein) and *gelE* (gelatinase). Among the screened virulence genes, *gelE* was the most frequent (32/79; 40.5%), followed by *cylA* (15/79; 19.0%) and *esp* (6/79; 7.6%). Details of the virulence genes identified in each isolate are provided in Supplementary Table S1.

The *gelE* gene was detected in 16/31 (51.6%) animal and 16/48 (33.3%) human isolates. The percentage of *gelE* in animal enterococci is consistent with the literature for animal isolates and higher than reported in humans, but *cylA* and *esp* levels were lower than previously observed [11,22,24].

The *cylA* gene was present in all β-hemolytic enterococci from companion animals, whereas in humans it was found in 10/15 (66.6%) of the β-hemolytic isolates. Other authors also reported a correlation between the phenotypic detection of hemolysis and the genotypic confirmation by *cylA* or *cylB* presence [21,22]. This result was expected, as *cylA* encodes a component of the cytolysin operon responsible for its activation and has been widely recognized as a molecular marker for hemolysin production in enterococci [59]. Moreover, this result supports the reliability of combining phenotypic and genotypic methods to study hemolysin production. In addition, this result is higher compared to a report of only 9% *cylA* gene prevalence among healthy human enterococci [24].

The *esp* gene was identified exclusively in companion animal isolates 6/31 (19.4%), and absent among human enterococci, which is not in line with the literature [24].

The *agg* gene was not detected in any group. A previous report found this gene to be present mainly in *E. faecalis* isolated from healthy dogs [22].

The moderate to high prevalence of virulence factors in commensal enterococci may represent a survival strategy, enhancing genetic diversity and thereby increasing their ability to persist within the host [24].

### 2.5. Comparison Between RAPD Profiling and Pathogenicity Patterns

**Figure 4:**
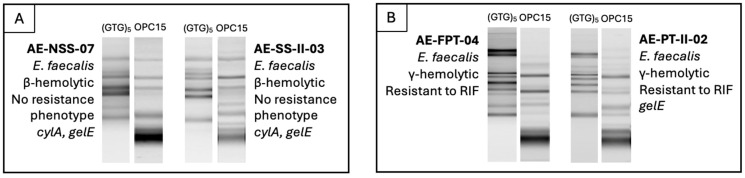
Comparison of humans from the same households (examples (**A**,**B**)), including RAPD profiles, species identity, hemolytic activity, antimicrobial resistance phenotypes, and virulence genotypes.

To address *Enterococcus* similarity from companion animals, humans, and animals and humans co-habiting the same household, RAPD-PCR profiles generated with (GTG)_5_ and OPC15 primers were analyzed alongside species identification, hemolytic ability, resistance phenotypes and virulence genes. Two similar RAPD profiles, with identical pathogenic-associated characteristics, were identified among humans within the same household (Figure 4), which can indicate shared colonization patterns among the same species and suggest host-specificity.

Recent research investigating the gut microbiota of humans and their companion animals has shown that pet ownership can alter the human gut microbiome, suggesting that animals may contribute to microbial transfer and enhance household microbial diversity [60,61,62]. Moreover, these microbiome shifts have been associated with a reduction in pathogenic bacteria alongside an increase in beneficial bacteria [60], being also linked to a lower risk of metabolic diseases [63]. Conversely, considerable interindividual microbiome variations have been described among companion animals living in the same household, particularly between species, supporting the view that each animal has its unique microbiome [62,64]. Accordingly, in this study, no comparable genetic or pathogenic-associated profiles were identified among animals from the same household. In this context, it is important to highlight that RAPD profiling, despite being successfully used to screen *Enterococcus* genetic similarity, has lower discriminatory power and reproducibility compared to other genotyping methods, such as MLST or PFGE, and can therefore only suggest genetic relatedness between isolates [65,66]. Confirmation of clonality and transmission between populations requires higher-resolution approaches such as whole-genome sequencing (WGS).

As previously noted, since only genetically distinct enterococci were selected for characterization rather than clonally matched within households, direct assessment of antimicrobial resistance dissemination was not possible, representing a limitation of our study design. In addition, the small sample size and number of participant households, geographical restriction and cross-sectional nature of the study design represent additional limitations. These factors reduce the diversity of environmental and social conditions that can influence antimicrobial resistance and virulence determinants dissemination, and they may be insufficient to detect bacterial exchange patterns between groups and households. Considering these limitations, our findings should be interpreted as an exploratory surveillance of prevalence and associated resistance patterns in companion animals and their human tutors.

Future research will focus on a thorough analysis of mobile genetic elements, horizontal gene transfer, and clonal relatedness of potential enterococcal links between animals and humans, providing insights into how enterococci and other commensal bacteria can contribute to interspecies dissemination of antimicrobial resistance across One Health settings. This study established a foundation for integrative genomic and epidemiological approaches that can explore the role of the household as an ecosystem in which commensal bacteria may act as reservoirs for the spread of pathogenic determinants across species.

## 3. Materials and Methods

### 3.1. Sampling

Fecal samples were collected from healthy companion animals and their human tutors between November 2022 and March 2023. A total of 45 fecal samples (*n* = 29 from animals; *n* = 16 from humans) were obtained from 5 households dispersed in the Lisbon metropolitan area (<10 km between each other), Portugal. The households included a total of 17 domestic animals (12 cats; 5 dogs) and 11 human tutors. Household A included 2 humans (ages 27–29) and 1 cat; household B, 3 humans (ages 5–46) and 3 cats; household C, 1 human (age 27), 2 dogs, and 2 cats; household D, 2 humans (ages 27–53), 3 dogs, and 3 cats; and household E, 3 humans (ages 30–52) and 3 cats. Household members were related through family relationships, including spouses, partners, or parent–child relations. Whenever possible, sampling was performed simultaneously but independently across households, with samples being collected from all available hosts on the same day to ensure paired human–animal representation.

For animals housed in groups (e.g., cats sharing a litter box), composite fecal samples were collected directly from the litter box, thoroughly homogenized, and processed as a single sample representing the group. All human fecal samples were self-collected by volunteer tutors using sterile containers and immediately stored at 4 °C until transport to the laboratory within 12 h of collection. Upon arrival at the laboratory, each sample was coded by household and host type (animal or tutor) and processed individually. To minimize bias and avoid transient selective pressures on antimicrobial resistance in commensal enterococci, neither animals nor humans had received antimicrobial treatment in the six months preceding sampling.

### 3.2. Microbial Isolation

After sample collection, individual samples or composites were diluted (1:10) in Maximum Recovery Broth (MRB; Liofilchem, Roseto degli Abruzzi, Italy), homogenized and incubated at 37 °C ± 2 °C for 24 h for enrichment. Subsequently, 100 μL of the enriched suspension were spread onto Slanetz and Bartley agar (SBA; Liofilchem, Roseto degli Abruzzi, Italy) and SBA supplemented with vancomycin (SBAvan; Liofilchem, Roseto degli Abruzzi, Italy) at 6 μg/mL, and incubated aerobically at 37 °C ± 2 °C for up to 48 h. Approximately 20% of characteristic colonies were randomly collected and further streaked onto Bile Esculin Azide agar (Liofilchem, Roseto degli Abruzzi, Italy) for presumptive *Enterococcus* genus confirmation. All presumptive *Enterococcus* isolates were maintained on Brain Heart Infusion (BHI; Liofilchem, Roseto degli Abruzzi, Italy) agar at 4 °C for routine use, and preserved in BHI broth with 20% glycerol at –80 °C.

### 3.3. Molecular Characterization

#### 3.3.1. DNA Extraction

Genomic DNA was extracted from pure cultures using the boiling method [67]. Briefly, a single colony was suspended in 50 μL of Tris-EDTA buffer containing 0.1% (*v*/*v*) Tween 20 (Merck KGaA, Darmstadt, Germany), and the bacterial suspension was incubated at 100 °C for 10 min. Immediately after incubation, the samples were placed on ice for 5 min to induce thermal shock and centrifuged at 13,000× *g* rpm for 5 min using Eppendorf^®^ 5415R centrifuge (Eppendorf, Hamburg, Germany). The resulting supernatant was directly used for PCR amplification and stored at −20 °C for complementary assays.

#### 3.3.2. Genetic Comparison

Genetic typing was carried out by RAPD-PCR using primers (GTG)_5_ (5′-GTGGTGGTGGTGGTG-3′) and OPC15 (5′-GACGGATCAG-3′) in independent procedures [68]. The reaction mixtures for each primer, with a total volume of 20 μL, contained 10 μL of NZYTaq II 2x Green Master Mix (NZYTech, Lisboa, Portugal) including NZYTaq II DNA polymerase, reaction buffer, deoxyribonucleotides (dNTPs), magnesium chloride (MgCl_2_), and additives; 7 μL of Milli-Q water (Millipore, Merck KGaA, Darmstadt, Germany), 2 μL of DNA and 1.0 μL of primer at 50 pmol/μL (STABVIDA, Caparica, Portugal).

Amplification was performed on a thermocycler GTC96S (Cleaver Scientific, Rugby, UK), under the following conditions: 94 °C for 5 min, followed by 40 cycles consisting of 94 °C for 1 min, annealing at 40 °C for 2 min, extension at 72 °C for 2 min, and a final extension at 72 °C for 10 min.

Genetic profiles were resolved on 1.2% agarose gel electrophoresis (GeneOn, Groß-Rohrheim, Germany) with 0.5× TBE buffer (4.5 × 10^−2^ M Tris, 4.5 × 10^−2^ M boric acid, and 1 × 10^−3^ M Na_2_EDTA, NYZTech, Lisboa, Portugal). Wells were loaded with 8 μL of amplification mixture, 2 μL of GelRed 10× (Biotium, Fremont, CA, USA), and 2 μL of bromophenol blue 6× (Merck KGaA, Darmstadt, Germany). Electrophoresis was performed at 90 V for 3 h. Gel results were observed in ChemiDoc XRS+ device (Bio-Rad, Hercules, CA, USA) with Image Lab 6.1. software (Bio-Rad, Hercules, CA, USA). Reproducibility was determined by 10% replicates, selected at random.

##### Data Analysis

Genetic profiles were analyzed using the BioNumerics software (version 6.6.5, BioMérieux, Marcy-l’Étoile, France). All gel images were normalized, and dendrograms generated based on Pearson correlation coefficients using the unweighted pair group method with arithmetic mean (UPGMA) with 3% tolerance. These dendrograms were used to select genomically distinct enterococci for further characterization, considering reproducibility level, isolates’ origin and household representativity. Reproducibility was assessed as the average similarity value observed between replicate profiles, with a threshold of 85% being set as the cut-off criterion.

#### 3.3.3. Species Allocation

Species and genus identification were carried out by PCR amplification, using primers FL1/FL2 for *E. faecalis*, FM1/FM2 for *E. faecium*, MUR1/MUR2 for *E. hirae*, DU1/DU2 for *E. durans*, and Ent1/Ent2 for *Enterococcus* genus, as listed in Table 5. The reaction mixtures for each set of primers, with a total volume of 20 μL, contained 10 μL of NZYTaq II 2x Green Master Mix (NZYTech, Lisboa, Portugal) including NZYTaq II DNA polymerase, reaction buffer, deoxyribonucleotides (dNTPs), magnesium chloride (MgCl_2_), and additives; 7 μL of Milli-Q water (Millipore, Merck KGaA, Darmstadt, Germany), 2 μL of DNA and 0.5 μL of forward and reverse primer at 50 pmol/μL (STABVIDA, Caparica, Portugal).

Positive controls (*E. faecalis* ATCC^®^ 29212, *E. faecium* E300, *E. hirae* DSMZ^®^ 20160, and *E. durans* DSM^®^ 20633) and negative controls (sterile water) were included in all reactions. Additionally, 10% of the isolates were tested as independent replicates to ensure reproducibility.

Amplification was performed on a thermocycler GTC96S (Cleaver Scientific, Rugby, UK), under the following conditions: 95 °C for 5 min; 35 cycles of 95 °C for 45 s, 57 °C for 45 s, and 72 °C for 45 s; followed by a final extension at 72 °C for 10 min.

Genetic profiles were resolved by electrophoresis on 1.2% agarose gels (GeneOn, Groß-Rohrheim, Germany) prepared with 0.5× TBE buffer (4.5 × 10^−2^ M Tris, 4.5 × 10^−2^ M boric acid, and 1 × 10^−3^ M Na_2_EDTA, NYZTech, Lisboa, Portugal). Each well was loaded with 8 μL of PCR product, 2 μL of GelRed™ (10×; Biotium, Fremont, CA, USA), and 2 μL of 6× bromophenol blue (Merck KGaA, Darmstadt, Germany). Electrophoresis was carried out at 90 V for 2 h, and gels were visualized using a ChemiDoc™ XRS+ imaging system with Image Lab 6.1. software (Bio-Rad, Hercules, CA, USA).

### 3.4. Antimicrobial Resistance

#### 3.4.1. Antimicrobial Susceptibility Testing

Antimicrobial susceptibility testing was evaluated using 16 clinically relevant antimicrobials, through the Kirby-Bauer disk diffusion method and following the Clinical and Laboratory Standards Institute (CLSI) guidelines M100 [32], VET01S [73] for *Enterococcus* spp. and VET01S for enrofloxacin using *Streptococcus* spp. values, as there are no breakpoints defined for enterococci. Briefly, each bacterial culture was aerobically grown overnight on rich media, suspended on sterile Ringer solution (Oxoid, Hampshire, UK) to a concentration of 0.5 in the MacFarland scale and spread plated. Antibiotic disks (Liofilchem, Roseto degli Abruzzi, Italy) were equidistantly placed on the agar surface, and the plates were incubated aerobically for 18–24 h at 37 ± 2 °C. The inhibition zones were measured and interpreted according to CLSI breakpoints. Multidrug resistance was defined as the acquired non-susceptibility to at least 1 antimicrobial in 3 or more antimicrobial classes [56].

The following antimicrobial list was selected considering its relevance in both human and veterinary practice as a treatment option for enterococcal infections: high-level gentamicin (120 μg), high-level streptomycin (300 μg), rifampicin (5 μg), enrofloxacin (5 μg), levofloxacin (5 μg), teicoplanin (30 μg), vancomycin (30 μg), erythromycin (15 μg), nitrofurantoin (300 μg), linezolid (30 μg), amoxicillin-clavulanic acid (30 μg), ampicillin (10 μg), chloramphenicol (30 μg), quinupristin-dalfopristin (15 μg), doxycycline (30 μg), tetracycline (30 μg) [11,56,74].

Agar screening and MIC determination were performed for enterococci reported as vancomycin resistant by the disk diffusion method and followed CLSI guidelines for dilution methods [32]. *Staphylococcus aureus* ATCC^®^ 25923 and *E. faecalis* ATCC^®^ 29212 were used as quality control for disk diffusion and MIC determination assays, respectively.

#### 3.4.2. Screening for Resistance Genetic Determinants

The antimicrobial resistance reported in antimicrobial susceptibility testing, to erythromycin, high-level gentamicin, tetracycline and doxycycline and vancomycin was confirmed by multiplex PCR, using the primers described in Table 6. Resistance genes were selected based on resistance phenotypes frequently observed in enterococci and on the clinical relevance of the corresponding antibiotics in both human and veterinary medicine.

The reaction mixtures contained a total volume of 20 μL, with 10 μL of NZYTaq II 2x Green Master Mix (NZYTech, Lisboa, Portugal) including NZYTaq II DNA polymerase, reaction buffer, deoxyribonucleotides (dNTPs), magnesium chloride (MgCl_2_), and additives; 2 μL of DNA, 0.5 μL of each forward and reverse primer at 50 pmol/μL (STABVIDA, Caparica, Portugal) and Milli-Q water (Millipore, Merck KGaA, Darmstadt, Germany). Positive (*E. faecalis* MMH 594, *E. faecalis* V583 or internal strain) and negative (sterile water) controls were included in all reactions. Additionally, 10% of the isolates were tested as independent replicates.

Amplification conditions were as follows: 95 °C for 5 min, 35 cycles of 95 °C for 45 s, 55 °C for 45 s, 72 °C 1 min; 72 °C for 5 min.

An aliquot of 8 μL of the amplification products was combined with 2 μL of GelRed™ 10× (Biotium, Fremont, CA, USA) and 2 μL of 6× bromophenol blue (Merck KGaA, Darmstadt, Germany), and the mixtures were electrophoresed on 1.2% agarose gel (GeneOn, Groß-Rohrheim, Germany) at 90 V for 2 h. Gels were visualized using a ChemiDoc™ XRS+ imaging system with Image Lab 6.1. software (Bio-Rad, Hercules, CA, USA).

For each antimicrobial class, the percentage of resistance gene detection was calculated using as denominator the total number of isolates resistant to that antimicrobial by disk diffusion, within each category (e.g., host origin or species).

### 3.5. Virulence Traits

#### 3.5.1. Hemolytic Activity

Briefly, metabolically active cultures were inoculated onto Columbia agar plates supplemented with 5% horse blood (Frilabo, Trofa, Portugal), and incubated under anaerobic conditions using Oxoid Anaerogen™ (Thermo Fisher Scientific, Waltham, MA, USA) sachets, for 48 h at 37 °C. After incubation, a transparent halo surrounding the colonies was interpreted as a positive reaction (β-hemolysis), a greenish halo as partial hemolysis (α-hemolysis), and the absence of any change in the medium as non-hemolytic activity (γ-hemolysis) (Teresa Semedo et al., 2003 [58]).

Reference strains *E. faecalis* MMH594 and *E. faecalis* ATCC 29212 were used as positive and negative controls, respectively. Reproducibility was ensured by performing 10% replicates, chosen at random.

#### 3.5.2. Screening for Virulence Genetic Determinants

Virulence gene screening was carried out by multiplex PCR (Table 7), targeting genes associated with cell aggregation (*agg*), immune evasion (*esp*), gelatinase production (*gelE*), and cytolysin/hemolysin activity (*cylA*).

The reaction mixtures for PCR contained a total volume of 20 μL, consisting of 10 μL of NZYTaq II 2x Green Master Mix (NZYTech, Lisboa, Portugal) including NZYTaq II DNA polymerase, reaction buffer, deoxyribonucleotides (dNTPs), magnesium chloride (MgCl_2_), and additives; 2 μL of DNA, 0.5 μL of each forward and reverse primer at 50 pmol (STABVIDA, Caparica, Portugal) and Milli-Q water (Millipore, Merck KGaA, Darmstadt, Germany). Positive (*E. faecalis* MMH 594) and negative (sterile water) controls were included in all reactions. Additionally, 10% of the isolates were tested as independent replicates. Thermocycler reaction conditions were as follows: 95 °C for 5 min, 35 cycles of 95 °C for 45 s, 55 °C for 45 s, 72 °C 45 s; 72 °C for 5 min.

An aliquot of 8 μL of the PCR products was mixed with 2 μL of GelRed™ (10×; Biotium, Fremont, CA, USA) and 2 μL of 6× bromophenol blue Merck KGaA, Darmstadt, Germany), and subsequently resolved on a 1.2% agarose gel (GeneOn, Groß-Rohrheim, Germany) by electrophoresis at 90 V for 2 h. Visualization was performed using a ChemiDoc™ XRS+ imaging system with Image Lab 6.1. software (Bio-Rad, Hercules, CA, USA).

### 3.6. Statistical Analysis

Pearson’s chi-squared test and Fisher’s exact test were used to evaluate statistically significant associations between bacterial species and host origin (companion animals vs. tutors), and antimicrobial resistance between host origin, species and household origin. For each antibiotic, resistance proportions between animals and tutors were compared using Pearson’s chi-squared test when expected cell counts were ≥5, otherwise Fisher’s exact test was used. Effect sizes were reported as odds ratios of tutors and animals with Haldane–Anscombe 95% CIs. Associations between resistance and household were tested for each antibiotic using Fisher’s exact test. *p*-values were adjusted across antibiotics through Benjamini–Hochberg correction to control false discovery rate in multiple comparisons.

The significance level (α) was set at 0.05. Statistical analyses were conducted using RStudio^®^ (version 2025.09.0+387), and both the database and the script used are provided as Supplementary Files.

## 4. Conclusions

This study highlights the role of companion animals as reservoirs of antimicrobial resistance and virulence within household environments, using *Enterococcus* as sentinel organisms. As this work was based on cross-sectional data collected between November 2022 and March 2023, temporal variations could not be evaluated. Enterococci were successfully recovered from fecal samples of both companion animals and humans (animal tutors), with *E. faecalis* identified as the predominant species in both hosts, followed by *E. faecium*. Distinct host-associated resistance profiles were observed: animal enterococci exhibited high rates of ampicillin resistance and multidrug resistance, while both groups showed comparable rates of erythromycin resistance. The detection of resistance genes such as *erm*(*B*) and *tet*(*M*), along with virulence determinants including *gelE and cylA*, further underscores the pathogenic potential of the microorganisms recovered from household settings. Although direct transmission was not proven, the shared resistance and virulence characteristics in household environments highlight the need for enhanced cross-species monitoring as potential contributors to the dissemination of antimicrobial resistance and virulence in integrated One Health contexts. Future studies will prioritize the analysis of mobile genetic elements, horizontal gene transfer, and clonal relatedness to clarify enterococcal transmission pathways and antimicrobial resistance dissemination across animal–human One Health interfaces.

## Figures and Tables

**Table 1:** Frequency of species among enterococci isolated from animals and humans.

Source	*E. faecalis*, *n* (%)	*E. faecium*, *n* (%)	*E. hirae*, *n* (%)	*Enterococcus* sp., *n* (%)
Companion animals (*n* = 31)	19 (61.2)	9 (29.0)	3 (9.7)	0 (0)
Tutors (*n* = 48)	25 (52.1)	17 (35.4)	4 (8.3)	2 (4.2)
Total (*n* = 79)	44 (55.7)	26 (32.9)	7 (8.9)	2 (2.5)

**Table 2:** Antimicrobial susceptibility results for enterococci from companion animals and their human tutors, showing frequencies of resistant, intermediate, and susceptible isolates.

Antimicrobials Classes and Agents		Resistant *n* (%)			Intermediate *n* (%)			Susceptible *n* (%)		
Class	Agent	Animals *n* = 31	Tutors *n* = 48	Total *n* = 79	Animals *n* = 31	Tutors *n* = 48	Total *n* = 79	Animals *n* = 31	Tutors *n* = 48	Total *n* = 79
Aminoglycosides	CN	0 (0.0)	1 (2.1)	1 (1.3)	0 (0.0)	0 (0.0)	0 (0.0)	31 (100)	47 (97.9)	78 (98.7)
	S	3 (9.7)	0 (0.0)	3 (3.8)	0 (0.0)	2 (4.2)	2 (2.5)	28 (90.3)	46 (95.8)	74 (93.7)
Ansamycins	RIF	17 (56.6)	13 (27.1)	30 (38.5)	4 (13.3)	7 (14.6)	11 (14.1)	9 (30.0)	28 (58.3)	37 (47.4)
Fluoroquinolones	ENR	1 (3.2)	0 (0.0)	1 (1.3)	18 (58.1)	15 (31.2)	33 (41.8)	12 (38.7)	33 (68.8)	45 (57)
	LEV	0 (0.0)	0 (0.0)	0 (0.0)	0 (0.0)	0 (0.0)	0 (0.0)	31 (100)	48 (100)	79 (100)
Glycopeptides	TEC	0 (0.0)	0 (0.0)	0 (0.0)	0 (0.0)	0 (0.0)	0 (0.0)	31 (100)	48 (100)	79 (100)
	VA *	16 (53.3)	0 (0.0)	16 (20.5)	10 (33.3)	2 (4.2)	12 (15.4)	4 (13.3)	46 (95.8)	50 (64.1)
Macrolides	E	9 (29.0)	14 (29.2)	23 (29.1)	15 (48.4)	14 (29.2)	29 (36.7)	7 (22.6)	20 (41.7)	27 (34.2)
Nitrofurans	F	1 (3.2)	0 (0.0)	1 (1.3)	1 (3.2)	0 (0.0)	1 (1.3)	29 (93.5)	48 (100)	77 (97.5)
Oxazolidinones	LZD	0 (0.0)	0 (0.0)	0 (0.0)	0 (0.0)	0 (0.0)	0 (0.0)	31 (100)	48 (100)	79 (100)
Penicillins	AMC	0 (0.0)	0 (0.0)	0 (0.0)	0 (0.0)	0 (0.0)	0 (0.0)	31 (100)	48 (100)	79 (100)
	AMP	31 (100)	0 (0.0)	31 (39.2)	0 (0.0)	0 (0.0)	0 (0.0)	0 (0.0)	48 (100)	48 (60.8)
Phenicols	C	0 (0.0)	3 (6.2)	3 (3.8)	0 (0.0)	0 (0.0)	0 (0.0)	31 (100)	45 (93.8)	76 (96.2)
Streptogramins	QDA ^a^	20 (64.5)	19 (39.6)	39 (49.4)	5 (16.1)	7 (14.6)	12 (15.2)	6 (19.4)	22 (45.8)	28 (35.4)
Tetracyclines	DXT	3 (9.7)	0 (0.0)	3 (3.8)	3 (9.7)	2 (4.2)	5 (6.3)	25 (80.6)	46 (95.8)	71 (89.9)
	TE	9 (29.0)	7 (14.6)	16 (20.3)	1 (3.2)	13 (27.1)	14 (17.7)	21 (67.7)	28 (58.3)	49 (62.0)

Legend: By alphabetic order of antimicrobial classes: CN—gentamicin, S—streptomycin, RIF—rifampicin, ENR—enrofloxacin, LEV—levofloxacin, TEC—teicoplanin, VA—vancomycin, E—erythromycin, F—nitrofurantoin, LZD—linezolid, AMC—amoxicillin-clavulanic acid, AMP—ampicillin, C—chloramphenicol, QDA—quinupristin-dalfopristin, DXT—doxycycline, TE—tetracycline. Notes: Percentages were calculated as the proportion of enterococci classified as resistant, intermediate, or susceptible relative to the total number of isolates tested for each antimicrobial, separately for animal, tutor and respective totals. * Vancomycin-resistant isolates by disk diffusion reported in this table were confirmed by dilution methods and were subsequently reclassified as susceptible. ^a^—Quinupristin-dalfopristin antimicrobial susceptibility results include *E. faecalis*, which is intrinsically resistant to this antibiotic.

**Table 3:** Antimicrobial resistance in enterococci from companion animals (*n* = 31) and their human tutors (*n* = 48), by species (*E. faecalis*, *E. faecium*, *E. hirae*, *Enterococcus* sp.).

Source	Species	Antibiotic, *n* Resistance (%)															
		CN	S	RIF	ENR	LEV	TEC	VA	E	F	LZD	AMC	AMP	C	QDA *	DXT	TE
Companion animals, *n* = 31	*E. faecalis* (*n* = 19)	0 (0.0)	3 (15.8)	9 (47.4)	0 (0.0)	0 (0.0)	0 (0.0)	0 (0.0)	7 (36.8)	0 (0.0)	0 (0.0)	0 (0.0)	19 (100)	0 (0.0)	19 (100) *	3 (15.8)	8 (42.1)
	*E. faecium* (*n* = 9)	0 (0.0)	0 (0.0)	6 (75.0)	1 (11.1)	0 (0.0)	0 (0.0)	0 (0.0)	2 (22.2)	1 (11.1)	0 (0.0)	0 (0.0)	9 (100)	0 (0.0)	0 (0.0)	0 (0.0)	1 (11.1)
	*E. hirae* (*n* = 3)	0 (0.0)	0 (0.0)	2 (66.7)	0 (0.0)	0 (0.0)	0 (0.0)	0 (0.0)	0 (0.0)	0 (0.0)	0 (0.0)	0 (0.0)	3 (100)	0 (0.0)	1 (33.3)	0 (0.0)	0 (0.0)
	Total	0 (0.0)	3 (9.7)	17 (56.7)	1 (3.2)	0 (0.0)	0 (0.0)	0 (0.0)	9 (29.0)	1 (3.2)	0 (0.0)	0 (0.0)	0 (0.0)	0 (0.0)	20 (64.5)	3 (9.7)	9 (29.0)
Tutors, *n* = 48	*E. faecalis* (*n* = 25)	1 (4.0)	0 (0.0)	5 (20.0)	0 (0.0)	0 (0.0)	0 (0.0)	0 (0.0)	3 (12.0)	0 (0.0)	0 (0.0)	0 (0.0)	0 (0.0)	2 (15.8)	17 (68.0) *	0 (0.0)	5 (20.0)
	*E. faecium* (*n* = 17)	0 (0.0)	0 (0.0)	8 (47.1)	0 (0.0)	0 (0.0)	0 (0.0)	0 (0.0)	10 (58.8)	0 (0.0)	0 (0.0)	0 (0.0)	0 (0.0)	0 (0.0)	0 (0.0)	0 (0.0)	1 (5.9)
	*E. hirae* (*n* = 4)	0 (0.0)	0 (0.0)	0 (0.0)	0 (0.0)	0 (0.0)	0 (0.0)	0 (0.0)	0 (0.0%)	0 (0.0)	0 (0.0)	0 (0.0)	0 (0.0)	0 (0.0)	0 (0.0)	0 (0.0)	0 (0.0)
	*Enterococcus* sp. (*n* = 2)	0 (0.0)	0 (0.0)	0 (0.0)	0 (0.0)	0 (0.0)	0 (0.0)	0 (0.0)	1 (50)	0 (0.0)	0 (0.0)	0 (0.0)	0 (0.0)	1 (50)	2 (100)	0 (0.0)	1 (50.0)
	Total	1 (2.1)	0 (0.0)	13 (27.1)	0 (0.0)	0 (0.0)	0 (0.0)	0 (0.0)	14 (29.2)	0 (0.0)	0 (0.0)	0 (0.0)	0 (0.0)	3 (6.2)	19 (39.6)	0 (0.0)	7 (14.6)

Antibiotics tested, by alphabetic order of classes: CN—gentamicin, S—streptomycin, RIF—rifampicin, ENR—enrofloxacin, LEV—levofloxacin, TEC—teicoplanin, VA—vancomycin, E—erythromycin, F—nitrofurantoin, LZD—linezolid, AMC—amoxicillin-clavulanic acid, AMP—ampicillin, C—chloramphenicol, QDA—quinupristin-dalfopristin, DXT—Doxycycline, TE—Tetracycline. * Quinupristin-dalfopristin antimicrobial susceptibility results include *E. faecalis*, which is intrinsically resistant to this antibiotic.

**Table 4:** Resistance gene profiles and phenotype–genotype comparison in companion animals and tutor enterococci. Percentages were calculated considering the total of isolates with a resistance phenotype to that antimicrobial/class, per category.

Antimicrobial or Class	Resistance Gene	Companion Animals n_genotype_/n_phenotype_ (%)	Tutors n_genotype_/n_phenotype_ (%)	Total n_genotype_/n_phenotype_ (%)
Gentamicin (high-level)	*aacA-aphD*	0 (0)	1/1 (100)	1/1 (100)
Erythromycin	*erm*(*B*)	4/9 (44.4)	3/14 (21.4)	7/23 (30.4)
Tetracyclines	*tet*(*M*)	5/9 (55.6)	0/7 (0)	5/16 (31.2)
Vancomycin	*vanA*	0/16 * (0)	0/0 (0)	0/16 (0)
	*vanB*			
	*vanC*			

* Vancomycin resistance reported by antimicrobial susceptibility testing was not confirmed by dilution and the isolates were therefore reclassified as susceptible. The reported number refers to enterococci considered resistant prior to confirmatory assays.

**Table 5:** Primers used for species and genus identification, including sequence and product length.

Identification	Primer	Primer Sequence (5′-3′)	Product Length (bp)	Reference
*E. faecalis*	FL1 FL2	F-ACTTATGTGACTAACTTAACC R-TAATGGTGAATCTTGGTTTGG	360	[69]
*E. faecium*	FM1 FM2	F-GAAAAAACAATAGAAGAATTAT R-TGCTTTTTTGAATTCTTCTTTA	215	[69]
*E. hirae*	MUR1 MUR2	F-CGTCAGTACCCTTCTTTTGCAGAGTC R-GCATTATTACCAGTGTTAGTGGTTG	521	[70]
*E. durans*	DU1 DU2	F-GCATTATTACCAGTGTTAGTGGTTG R-TGAATCATATTGGTATGCAGTCCG	186	[71]
*Enterococcus* spp.	Ent1 Ent2	F-TACTGACAAACCATTCATGATG R-AACTTCGTCACCAACGCGAAC	112	[72]

**Table 6:** Primers used for the identification of antimicrobial resistance genes, including sequence and product length.

Gene	Resistance to	Primer Sequence (5′-3′)	Product Length (bp)	Reference
*erm*(*B*)	Erythromycin	F-GAAAAGGTACTCAACCAAATA R-AGTAACGGTACTTAAATTGTTTAC	639	[75]
*aacA-aphD*	High-level gentamicin	F-GATTGCCAGAACATGAATTACACGA R-CATAACCACTACCGATTATTTCAAT	156	[76]
*tet*(*M*)	Tetracycline, doxycycline	F-ACAGAAAGCTTATTATATAAC R-TGGCGTGTCTATGATGTTCAC	155	[77]
*vanA*	Vancomycin	F-TTGGGGGTTGCTCAGAGGAG R-CTTCGTTCAGTACAATGCGG	931	[76]
*vanB*		F-AAGCTATGCAAGAAGCCATG R-CCGACAATCAAATCATCCTC	536	[78]
*vanC*		F-GCAGGTTCTGCCTTATGTATGAA R-ATGAAATGGCGTCACAAGCA	339	[76]

**Table 7:** *Enterococcus* virulence factors, including gene, biological role, primers, product length and reference.

Gene	Role in Virulence	Primer Sequence (5′-3′)	Product Length (bp)	Reference
*agg*	Aggregation protein with a role in adherence to eucaryotic cells and cell aggregation and conjugation	F-CGGTACAGTTGGCAGTGTTTCG R-GGCTTGTGGGTCTTTGGCAGAG	775	[79]
*gelE*	Extracellular metalloendopeptidase, hydrolyzes gelatin, collagen, hemoglobin and other compounds	F-ACCCCGTATCATTGGTTT R-ACGCATTGCTTTTCCATC	419	[80]
*cylA*	Activation of cytolysin which lyses a range of eukaryotic and Gram-positive cells	F-CGGGGATTGATAGGCTTCATCC R-TAACCATCTGGAAAGTCAGCAG	628	[79]
*esp*	Cell wall protein involved in immune evasion, which may be associated with *cyl* genes located on a pathogenicity island	F-5′ TTGCTAATGCTAGTCCACGACC R-5′ GCGTCAACACTTGCATTGCCGAA	933	[80]

## Data Availability

The original contributions presented in this study are included in the article/Supplementary Material. Further inquiries can be directed to the corresponding author.

## Supplementary Materials

The following supporting information can be downloaded at https://www.mdpi.com/article/10.3390/ijms27020654/s1.

## Abbreviations

The following abbreviations are used in this manuscript:

AMR	Antimicrobial resistance
ARGs	Antibiotic resistance genes
CLSI	Clinical and Laboratory Standards Institute
EUCAST	European Committee on Antimicrobial Susceptibility Testing
MDR	Multidrug-resistant
MIC	Minimum Inhibitory Concentration
MLST	Multi-locus sequence typing
MRB	Maximum recovery broth
PBP5	Penicillin-binding protein 5
PFGE	Pulsed-field gel electrophoresis
RAPD	Random Amplified Polymorphic DNA
SBA	Slanetz and Bartley agar
SBAvan	Slanetz and Bartley agar supplemented with vancomycin
UTIs	Urinary tract infections
VRE	Vancomycin-resistant enterococci
WGS	Whole genome sequencing
